# Knowledge, attitudes and misconceptions of primary care physicians regarding fever in children: a cross sectional study

**DOI:** 10.1186/1824-7288-38-40

**Published:** 2012-09-05

**Authors:** Figen Demir, Ozgur Sekreter

**Affiliations:** 1 Department of Public Health, Acıbadem University School of Medicine, Gülsuyu Mah. Fevzi Çakmak Cad. Divan Sok. No: 1, Maltepe/İSTANBUL, 34848, Turkey; 2 Department of Communicable Diseases, The Ministery of Health of Turkey Local Health Authority, Yayla Mah. Ömer Karahasan Sok. No:6, Zonguldak, 67500, Turkey

**Keywords:** Children, Fever, Physician attitudes

## Abstract

**Background:**

Fever is an extremely common sign in paediatric patients and the most common cause for a child to be taken to the doctor. The literature indicates that physicians and parents have too many misconceptions and conflicting results about fever management. In this study we aim to identify knowledge, attitudes and misconceptions of primary care physicians regarding fever in children.

**Methods:**

This cross-sectional study was conducted in April-May 2010 involving primary care physicians (n=80). The physicians were surveyed using a self-administered questionnaire. Descriptive statistics were used.

**Results:**

In our study only 10% of the physicians knew that a body temperature of above 37.2°C according to an auxiliary measurement is defined as fever. Only 26.2% of the physicians took into consideration signs and symptoms other than fever to prescribe antipyretics. 85% of the physicians prescribed antipyretics to control fever or prevent complications of fever especially febrile seizures. Most of the physicians (76.3%) in this study reported that the height of fever may be used as an indicator for severe bacterial infection. A great majority of physicians (91.3%) stated that they advised parents to alternate the use of ibuprofen and paracetamol.

**Conclusions:**

There were misconceptions about the management and complications of fever. There is a perceived need to improve the recognition, assessment, and management of fever with regards to underlying illnesses in children.

## Background

Fever is an extremely common sign in paediatric patients and the most common cause for a child to be taken to the doctor [1]. Fever in children less than five years of age can be a diagnostic challenge for primary care physicians and even for paediatricians, because it is often difficult to identify the cause. In most cases, fever is due to a self-limiting viral infection including acute upper respiratory infection, however, it may also be the presenting feature of serious bacterial infections such as meningitis or pneumonia, fortunately this represents a minority of cases [2]. In these cases fever is considered a beneficial part of the immune response [3,4]. More recent studies have shown that a significant number of children (around 48%) show no obvious cause of fever despite careful assessment [5]. Paediatricians recommend the natural way and/or prescribe medication to lower the fever. This paediatric policy is not intended to quicken recovery but to ensure the comfort of the child [6]. Actually there are only a few reasons for using the antipyretic therapy mentioned above [3,4]. For more than three decades the beneficial effects of mild fever have been known [7]. In 1980 Schmitt discussed parental misconceptions and fear of fever and defined this fear as fever phobia [7]. Physicians contribute to this fever phobia by their incomplete and insensible messages to parents. May and Baucher published a study conforming to the problem of misinformation about fever. This study set forth that instructions given to parents about fever are often inaccurate and physicians also have some misconceptions about fever and its complications. Most of the primary care physicians and even paediatricians believed that fever is dangerous and cause brain damage and febrile convulsions [8].

Although fever has beneficial effects and is good for the immune response, it seems that the negative perceptions of fever, like fears of febrile seizures and fever phobia, remain unchanged. Physicians continue to reduce low grade fever without other symptoms and recommend various kinds of antipyretics to feverish children despite initial treatment [9,10]. In brief, these misconceptions cause unnecessarily aggressive and inappropriate management of feverish children [7,11-16].

Nevertheless there are also conflicting results about fever management in the literature and these conflicts may also contribute to physicians’ misconceptions.

In this study we aim to identify knowledge, attitudes and misconceptions of primary care physicians regarding fever in children.

## Methods

This cross-sectional study was conducted in April-May 2010 involving all primary care physicians working in a province with a population of 600 000.

During vaccine distribution, researchers went to each primary health care centre to inform physicians about the study. Data was collected after the purpose of the study was explained to the participants and they were informed that their participation was voluntary. Approval for the study was obtained from the Turkish Ministry of Health Local Health Authority of the province in which the research was carried out. The study participants were all primary care physicians who worked in primary health care facilities during the period of study. A sample was not selected, but there were attempts to contact the whole population. Physicians who were on holiday or ill at home during the period of study were excluded from the study. Two of 82 physicians refused participation (the participation rate was 97.4%). Physicians were surveyed using a self-administered questionnaire. It was comprised primarily of closed ended questions about basic demographic characteristics, working conditions, number of working years and setting of practice, knowledge, attitude and management of fever in children.

The data was analyzed using the SPSS 16.0 program and descriptive statistics were used.

## Results

The study included 80 physicians (participation rate 97.4%) with a mean age of 36.5 ± 8.3 and the male–female ratio was 1. The socio-demographic characteristics of the participants are presented in Table 1.

**Table 1:** Demographical Characteristics of Physicians

**Characteristics**	**Descriptive statistics**
**Female, N (%)**	40 (% 50)
**Age (mean ± sd)**	36.5 ± 8.3
**Marital status**	
**Married n (%)**	60 (%75)
**Single n (%)**	20(%25)
**Participant with children**	
**Yes n (%)**	52 (%65)
**No n (%)**	28(35%)
**Duration of working (mean ± sd)**	10.8 ± 7.8
**Number of patients per week (mean ± sd)**	357.4 ± 188.1

*N* number, *sd* standard deviation.

There was an internet connection in all of the health care centres. Only a few physicians (7.5%) undertaken training regarding fever after their postgraduate studies and 27.5% of the physicians indicated that they had read an article associated with fever in the last 6 months.

Most of the physicians (83.8%) recommended an auxiliary measurement of fever to the parents of the febrile child and 10% of them indicated that a body temperature of above 37.2°C, according to an auxiliary measurement, was treated as fever. The body temperature treated as fever by physicians according to an auxiliary measurement varied between 36.5°C and 39°C. About two thirds of physicians (73.8%) reported that they recommended an antipyretic agent to every child under the age of 5 with fever. Only 26.2% of physicians took into consideration signs and symptoms other than fever (malaise, irritability, prolonged crying, signs of infection) to prescribe the antipyretic. Nevertheless only 15% of physicians indicated that they prescribed antipyretics to ensure the child’s comfort and remove irritability. The rest of the physicians prescribed antipyretics to control fever and prevent complications of fever, especially febrile seizures.

Some of the statements regarding the management and complications of fever, in agreement with these statements of physicians, are shown in Table 2.

**Table 2:** Distribution of physicians’ agreement to some statements regarding fever management and complications in febrile children under the age of 5

**Statements**	**Number**	**Percent**
**Fever is dangerous for a child**	52	65.0
**A fever lower than 38°C should definitely be treated even when there are no other signs and symptoms**	14	17.5
**A body temperature of above 38°C must definitely be treated whatever the underlying pathology**	56	70.7
**Prevention of febrile convulsion is the main reason for antipyretic usage**	60	75.0
**Brain damage, seizures and death are complications of fever**	62	77.5
**Medical treatment must definitely be used in reducing fever**	29	36.3
**Physical methods like baths should be recommended to reduce fever**	70	87.5
**Cold application should be recommended to reduce fever**	56	70.0
**Rubbing the body with alcohol must be recommended to reduce fever**	18	22.5
**Sleeping febrile children must not be disturbed**	8	10.0
**Fever is a risk factor for brain damage**	68	85.0
**The risk of febrile convulsion increases when the fever increases**	68	85.0
**Brain damage may occur after febrile convulsion**	72	90.0
**Teething is a reason for fever**	57	71.3
**Paracetamol or ibuprofen usage should be recommended to prevent fever and local reactions associated with childhood vaccination**	65	81.3
**High fever may be used as an indicator of severe bacterial infection**	61	76.3
**Paracetamol and ibuprofen are the only antipyretic drugs recommended for use in children**	53	66.3
**Ibuprofen and paracetamol can be used alternatively**	73	91.3
**Acetylsalicylic acid should not be used in a febrile child**	73	91.3
**Oral administration of paracetamol is preferable to rectal administration in children**	62	77.5

Most of the physicians (90%) indicated that febrile convulsions can cause brain damage. More than half (65.0%) of the physicians said that fever is harmful for the child and 70.7% of them reported that a body temperature of above 38°C must definitely be treated, whatever the underlying pathology . Many (76%) believed that the main reason for antipyretic usage is to prevent febrile convulsion and 87.5% indicated that physical methods (bathing) should be recommended to reduce fever. Other physical methods like cold application and rubbing the body with alcohol were also recommended by the majority of the physicians (Table 2). Most of the physicians (84%) believed there is a positive correlation between the height of fever and the incidence of febrile convulsion. According to 82.7% of the physicians, teething is a cause of fever. Inappropriate beliefs about antipyretics were confirmed by the 78.7% who agreed that paracetamol and ibuprofen can be used alternatively. More than half (68%) of the physicians agreed that only paracetamol and ibuprofen should be used as antipyretics in children.

## Discussion

The first major finding of this research is a variation in the definition of fever. Fever is defined as a body temperature greater than 37.2°C according to an auxiliary measurement by primary care physicians [10]. In our study only 10% of physicians knew that a body temperature of above 37.2°C according to an auxiliary measurement is defined as fever. Body temperature treated by physicians as fever varied between 36.5°C and 39.0°C. This range is too great. Definitions of high fever by physicians also varied significantly in other studies [17,18]. In our study most of the physicians (83.8%) recommended an auxiliary measurement of fever to the families. There are conflicting results as regards this subject. Some authors consider tympanic measurement the best method for non-invasive measurement [19-21], some authors recommend an auxiliary measurement because it is easy to perform and generally well tolerated. However, it is not very sensitive [22]. According to the Italian paediatric society guidelines, auxiliary measurements using a digital thermometer is recommended in all children for measurements taken at home [23]. Physical temperature reducing methods such as cold application and rubbing the body with alcohol were recommended in our study. But in fever management guidelines and studies of the use of these methods to reduce fever, physical methods are not recommended as their usage may be associated with adverse effects and a paradoxical increase in fever [23-26]. Severe complications such as hypoglycaemia, coma or even death may be seen in a febrile child due to rubbing with alcohol [24,25].

In the present study about two thirds of physicians (73.8%) recommended an antipyretic agent to every child under the age of 5 with fever, whatever the signs and symptoms. Only 26.2% of physicians took into consideration signs and symptoms other than fever (malaise, irritability, signs of infection) to prescribe antipyretic. In fact according to guidelines, antipyretics should not be used routinely in management of a febrile child [20,21]. Use of antipyretics in children is recommended in case of prolonged crying, irritability, reduced activity and sleeplessness [27].

Only 15% of physicians indicated that they prescribed antipyretics to ensure a child’s comfort and remove irritability, except for reducing fever. The rest of the physicians prescribed antipyretics to control fever, and prevent complications of fever especially febrile seizures. International literature confirms that fever phobia is common among parents and health care workers. Misconceptions about complications of fever especially febrile convulsions often push health care workers to over treat fever and this reinforces the phobia among parents [9,28]. The result of the present study confirmed these findings. According to our study there have been few changes in physicians’ knowledge and attitudes over the past years. Fever phobia continues. More than half of the physicians (65%) considered fever to be dangerous for a child. It is known that antipyretic treatment has not been effective in the prevention of simple febrile seizures [29-32]. In our study 75% of physicians reported that the main reason for antipyretic usage was to prevent febrile seizure. In another study which was conducted in Saudi Arabia, this ratio was 70%, i.e. similar to that of our study [9]. In Israel 8.7% of 1000 primary care physicians, paediatricians, general practitioners and family specialists considered avoidance of febrile seizure to be the main reason of antipyretic usage [33]. Due to the participation of specialists, the misconception was less common in this population.

Although there have been no evidence that fever causes brain damage unless it reaches above 41°C, it is still a common misconception among physicians [34,35]. Fortunately, fever seen in children rarely reaches this high temperature. The most common side effects of fever are benign and include minimal dehydration, increased sleepiness, and discomfort [36]. In our study 85% of physicians stated that fever was a risk factor for brain damage. There are also other articles that surveyed physicians’ opinions and behaviours with regards to fever, which confirm that fever is seen as a risk factor for brain damage. Unfortunately health care workers and parents believe that brain damage is a consequence of fever [34,35].

Febrile seizure is a rare complication of fever, occurs in 2-4% of febrile children and most are self-limited without any long-term sequelae [36-41]. Despite there being no evidence to suggest that brain damage may occur after febrile convulsion [42-44], in our study 90% of the physicians believed that brain damage might occur after febrile convulsion.

This misconception is common not only in our study population but also among other physicians working in primary health care, hospitals and emergency rooms [8,9,45].

In our study 85% of physicians agreed that the higher the temperature, the higher the likelihood of a febrile seizure. There are conflicting results about the association between the risk of febrile convulsion and the height of fever. Some authors think that height of body temperature plays a more important role in the pathogenesis of a febrile seizure than the rapidity of the rise in temperature [46-48], but some disagree with this observation [49]. It is said that the most significant risk factor for the development of a first febrile seizure is the height of the temperature; the higher the temperature, the higher the likelihood of a febrile seizure [50]. Nevertheless according to other authors; febrile seizures may be more likely to occur with rapid rises in temperature [51], at the onset of febrile illnesses or with a rapid decrease in temperature (alcohol sponging). In a study one third of all children who have febrile seizures will have a second episode despite attempts to prevent fever with antipyretics [52]. In a randomised controlled trial, 157 children who enrolled after their first febrile convulsion were followed for two years. In this study there was no evidence found that antipyretic treatment reduced the risk of febrile convulsions [53]. The last two observations supported the argument that height of fever may not be related to febrile seizures.

Most of the physicians (76.3%) in this study reported that height of fever can be used as an indicator for severe bacterial infection. Some studies have found a causal relationship between the height of fever and the severity of the underlying pathology e.g. bacterial infection; others have not [54-57]. According to evidence obtained from observational studies height of fever should not be taken as an indicator of the severity of the underlying pathology by itself. In children of less than 3 months of age, height of fever may be an indicator of severe bacterial infection [23].

In our study 10% of participants agreed that a sleeping febrile child should not be disturbed. There are studies which show that parents, physicians and nurses awaken sleeping febrile children who have no other symptoms for antipyretic administration [33,58,59]. According to some paediatricians, sleeping febrile child should not be awaken for any reason, including medication [9,33].

More than half of participating physicians (66.3%) agreed that paracetamol and ibuprofen are the only antipyretic drugs which should be used in children. Studies show that both drugs are more effective than placebo, and can be used confidently in children [60-63]. Evidence obtained from randomised controlled clinical trials show that paracetamol and ibuprofen are the only antipyretic drugs recommended for use in children [23].

Because of the risk of Reye’s syndrome, use of acetylsalicylic acid in children is not recommended [23]. Most of the physicians who participated in the present study (91.3%) reported that Acetylsalicylic acid should not be used in a febrile child, although this has been known for a long time, nearly 10% of physicians still disagreed.

In this study 77.5% of physicians preferred oral administration to rectal administration. Some investigations show that oral acetaminophen is more effective than the rectal form [64], others found they had similar effects [65,66] so the comparison of the antipyretic effects of rectal and oral acetaminophen has conflicting results. Use of rectal paracetamol is not recommended by the Italian Paediatric Society [23] because of the risk of overdose. It is difficult to achive precise dosage in rectal administration. It depends on the child’s body weight rather than age [67].

There are conflicting results about fever management in the literature and this situation could affect the practices of physicians. In many articles alternative treatments of fever with paracetamol and ibuprofen are recommended [68-72] and alternating acetaminophen and ibuprofen in febrile children appears to be a common practice among physicians [70]. But according to a guideline about management of fever in children; combined or alternating the usage of ibuprofen and paracetamol is not recommended [23]. There is no evidence available that alternating therapy results in improvement in other clinical outcomes and there is also no evidence regarding the safety of this practice [73-75]. In the present study a great majority of physicians (91.3%) stated that they advised parents to alternate the use of ibuprofen and paracetamol.

Preventive usage of antipyretic before vaccine application is a common implementation in primary health care in Turkey despite the absence of evidence . Most of the physicians (81.3%) in our study reported that antipyretic usage should be recommended to prevent fever and local reaction associated with childhood vaccination. According to evidence obtained from well-designed randomised clinical trials, use of paracetamol or ibuprofen is not recommended to reduce fever and local reactions associated with vaccination [76,77].

According to the present study, 71.3% of physicians reported that teething is a reason for fever. In a prospective study which was designed to clarify symptoms associated with teething, the most common symptoms were biting, drooling, gum-rubbing and sucking. Generalised irritability and a mild fever might also occur during the teething period [78]**.** In an another prospective study the variation in temperature remained within the normal range during the teething period [79]**.**

## Conclusions

Our data suggests that implementation of educational programs and using guidelines regarding the proper management of the febrile child are needed. There were misconceptions about management and complications of fever. Conflicting results about fever in the literature also confirm these misconceptions.

Although there are guidelines for many diseases in primary care, there is no national guidance on the management of fever in Turkey. Management varies across Turkey and also among physicians. As a result, there is a perceived need to improve the recognition, assessment, and management of fever with underlying illnesses in children.

## Competing interest

The authors declare that they have no competing interests.

## Authors’ contributions

FD, chose the subject, was responsible for the planning and conducting of the study, collected the data, analyzed data and was primarily responsible for the writing of the manuscript. OS, collected the data and was responsible for the planning of the study. All authors read and approved the final manuscript.
